# Obesity in K–7 Students — Anchorage, Alaska, 2003–04 to 2010–11 School Years

**Published:** 2013-05-31

**Authors:** Myde Boles, Clyde Dent, Karol Fink, Charles Utermohle, Andrea Fenaughty

**Affiliations:** Program Design and Evaluation Svcs, Multnomah County Health Dept and Oregon Public Health Div; Chronic Disease Prevention and Health Promotion, Alaska Dept of Health and Social Svcs

Childhood obesity is a major public health concern in the United States. National data indicate that from 1999 to 2010, obesity stopped increasing among females aged 2–19 years but continued to increase among males (1). Other reports have suggested that obesity is decreasing in certain geographic areas (2) or among certain groups of children (3). In the metropolitan area of Anchorage, Alaska, during the 2003–04 school year, an estimated 16.8% of children in the Anchorage and Matanuska-Susitna Borough school districts in grades K, 1, 3, 5, and 7 were obese, similar to a 2003–2006 national estimate of 17.0% for youths aged 6–11 years (4). To determine whether trends in the two Anchorage-area school districts mirror those in the rest of the United States, the Alaska Department of Health and Social Services analyzed body mass index (BMI) data for public schoolchildren in grades K, 1, 3, 5, and 7 for the 2003–04 to 2010–11 school years. This report summarizes the results of that analysis, which found that, overall, the prevalence of obesity decreased by 3.0% from 2003–04 to 2010–11, and the decline varied widely by subgroup. The decrease was significant among boys (5.5%), white students (15.1%), students in grades K, 1, and 3 (5.4%), and students in schools where ≤50% of students were receiving subsidized lunches (8.2%). Efforts are needed to further reduce the prevalence of childhood obesity in the Anchorage area and to focus on poorer schools and those groups with the highest prevalence of obesity.

School-based measurement of student height and weight to calculate BMI is widely accepted for obesity surveillance to assess prevalence, monitor trends, and evaluate outcomes of interventions (5). Nationally, 20 states require school districts to measure students’ height and weight (6). Alaska does not require school districts to measure height and weight; however, measurements are taken and recorded as part of routine health screenings in many Alaska school districts that employ school nurses. The findings in this report are based on data obtained by the Alaska Department of Health and Social Services from the two school districts in the Anchorage metropolitan statistical area: Anchorage School District (ASD) and Matanuska-Susitna Borough School District (MSBSD). The records include information on student height, weight, race/ethnicity, age, sex, and grade level. School-level information also was obtained on the proportion of students enrolled in the subsidized lunch program, which was used as a proxy for whether the school had lower or higher socioeconomic status (SES).

BMI of the students was analyzed overall and by sex, grade level, and race/ethnicity. Students were categorized as non-Hispanic American Indian/Alaska Native (AI/AN), non-Hispanic white, or as all other race/ethnicities. Data cleaning resulted in the exclusion of approximately 6% of the total measurements reported in both school districts, leaving 152,803 valid records, reflecting an average of 19,100 students measured each year. In ASD, height and weight measures accounted for 86% of total student enrollment in the represented grades during the 8-year period. In MSBSD, height and weight measurements were available for only 52% of total student enrollment in the represented grades during the 8 years because of a lack of school nurses and training. BMI percentiles for age and sex were calculated using growth charts from CDC (7). Obesity was defined as BMI ≥95th percentile for age and sex.

For each school year, the weighted sample estimates matched known population totals for enrollments per year by demographic categories for the two school districts (ASD and MSBSD), two grade-range categories (grades K, 1, and 3 and grades 5 and 7), sex (male and female), and race/ethnicity (white, AI/AN, and all other races/ethnicities). Population enrollment data were obtained from the National Center for Education Statistics of the U.S. Department of Education (8). For each school year during 2003–2009, analysis weights were defined as the ratio of population enrollments to the sample size obtained in each demographic category. Population data were not available for 2010; therefore, 2009 population data were used for 2010 calculations.

A multivariate logistic regression model was used to test for trend in obesity prevalence. The model included a linear term for time, along with sex, grade, race/ethnicity, school district, and SES. Schools with ≤50% of students in the subsidized lunch program were considered to have higher SES, and schools with >50% students in the subsidized lunch program lower SES. The model was stratified by sex, grade, school district, and SES to examine obesity trends in subgroups. In this report, all increases and decreases described are statistically significant unless otherwise indicated. A Pearson chi-square test was used to compare prevalence estimates between subgroups at a single point in time.

From 2003–04 to 2010–11, the overall prevalence of obesity in the two Anchorage-area school districts decreased by 3.0%, from 16.8% to 16.3% (Table). The prevalence of obesity among boys decreased 5.5%, from 18.1% to 17.1%, and the prevalence among white children decreased 15.1%, from 13.9% to 11.8%. The prevalence of obesity did not decrease among girls, AI/AN children, and children in other racial/ethnic groups. Among children in grades K, 1, and 3, the prevalence of obesity decreased 5.4%, from 14.8% to 14.0%; the prevalence did not decrease among children in grades 5 and 7. The prevalence of obesity among children in ASD schools decreased 2.2%, from 18.0% to 17.6%; the prevalence among children in MSBSD schools did not decrease but was significantly lower (12.5% in 2003–04 and 12.4% in 2004–05) than the prevalence observed in ASD schools (Table).

Among children in schools with higher SES, the prevalence of obesity decreased 8.2%, from 14.6% to 13.4%; the prevalence did not decrease among children in schools with lower SES. In 2010–11, by SES and grade level group, the highest prevalences of obesity by racial/ethnic group were among children in grades 5 and 7 in schools with lower SES: AI/AN (26.0%), white (22.4%), and all other racial/ethnic groups (31.1%) (Figure). In 2010–11, the prevalence of obesity was significantly higher (22.6%) among students in schools with lower SES than among students in schools with higher SES (13.4%) (Table).

## Editorial Note

The findings in this report indicate that, from 2003–04 to 2010–11, the prevalence of obesity among public school students in grades K–7 in the Anchorage metropolitan area decreased overall and within certain demographic subgroups. Declines in obesity prevalence were observed among children in schools with higher SES, boys, white children, and children in grades K, 1, and 3. No statistically significant decreases in obesity were observed among AI/AN or other racial/ethnic minorities. This report underscores the persistent differences in the prevalence of obesity among children of different race/ethnicities. Of particular concern is the continued high prevalence of obesity among AI/AN children.

Over the past decade, various practices, programs, and policies have been created in Alaska, by the state government and by ASD and MSBSD, to address childhood obesity. In 2002, the Alaska Department of Health and Social Services established its Obesity Prevention and Control Program. The following year, the program convened the first statewide Obesity Summit and initiated the Alaska-specific Obesity Prevention and Control Plan. In 2003, the program published its *Burden of Overweight and Obesity in Alaska* report (9), and later developed Alaska’s Statewide Physical Activity and Nutrition Plan.

The overall decline in obesity from 2003–04 to 2010–11 coincides with some obesity-related interventions in ASD and MSBSD. For example, in 2006, ASD adopted and implemented a wellness policy that banned the sale or provision of soda and junk food in vending machines, school stores, school and administrative offices, school cafeteria fountain drink machines, and fundraisers. In 2007–08, ASD adopted a revised elementary student schedule that increased health instruction, including nutrition education, by 30 minutes each week. The following year, the elementary student schedule was revised again to provide a 50% increase in physical education instruction. In 2005, MSBSD adopted a policy that set nutrition standards for any snack or beverage sold outside the federally subsidized meal program. In 2005–06, MSBSD designated a district wellness coordinator to maintain an ongoing active school wellness council that involved students, parents, food service personnel, school boards, school administrators, health professionals, and community members. Additional policies were adopted during 2005–2008 that included limiting the use of food as a reward, marketing only those foods and beverages that met nutrition standards, limiting physical education from being withheld for disciplinary purposes, and implementation of a universal, no-cost school breakfast policy.

The findings in this report are subject to at least one limitation. Height and weight measurements were not collected through a statistically valid sampling procedure, using regularly calibrated equipment, but were obtained as part of the routine school health screening process. However, because efforts were made to screen all students in grades K, 1, 3, 5, and 7, it is unlikely that the prevalence of overweight and obesity was subject to a bias that resulted in the disproportionate selection of more obese students. In addition, prevalence estimates were weighted to be representative of the entire enrollment for each year, further minimizing bias.

What is already known on this topic?

After many decades of increases in the prevalence of childhood obesity in the United States, recent reports indicate a stabilization of obesity prevalence in female children and adolescents nationally and a decline in obesity in selected areas of the country.

What is added by this report?

Childhood obesity prevalence among public school children in grades K, 1, 3, 5, and 7 in the metropolitan area of Anchorage, Alaska, decreased by 3.0%, from 16.8% in 2003–04 to 16.3% in 2010–11. Although obesity decreased significantly overall, the decline in obesity prevalence was not observed among children in all racial/ethnic groups, nor among children in schools serving children with lower socioeconomic status.

What are the implications for public health practice?

The lack of progress in reducing the prevalence of obesity among racial/ethnic minorities and children in schools with lower socioeconomic status highlights the need to implement targeted, culturally specific interventions to population subgroups to reduce childhood obesity.

The objectives of this study were to examine trends in obesity prevalence estimates that are representative of the public school population in the Anchorage metropolitan statistical area. Although a causal relationship cannot be inferred between the reduction in prevalence and school-based policies, programs, and practices implemented by ASD and MSBSD, the trend toward reduced prevalence of obesity is consistent with findings described in other reports (2,3). The declines in obesity prevalence among white children and children in schools with higher SES suggest that changes in the school environment, if they are effective in reducing obesity, might not be reaching all students. The lack of progress among AI/AN children and those of lower SES highlights the need for further targeted measures to reduce childhood obesity.

## Figures and Tables

**FIGURE:**
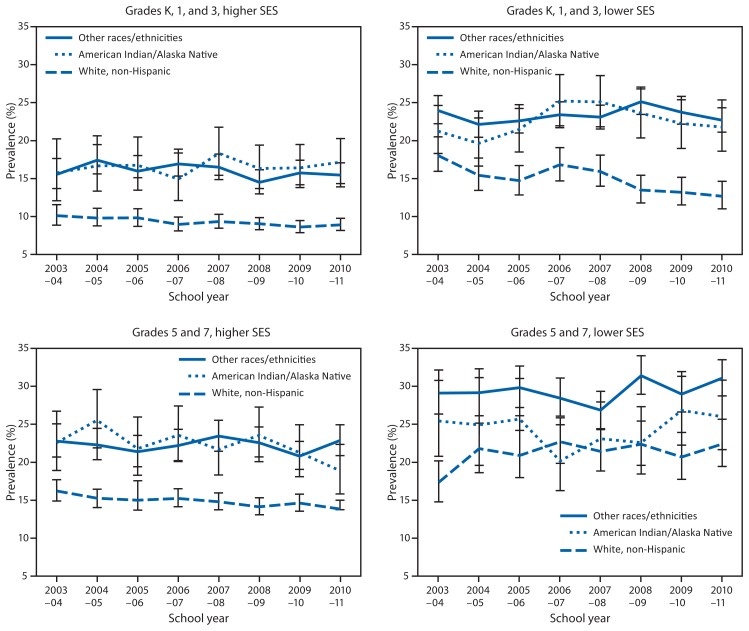
Prevalence of obesity^*^ among public school children in grades K, 1, 3, 5, and 7, by grade, race/ethnicity, and socioeconomic status (SES) — Anchorage metropolitan area, 2003–04 through 2010–11 school years (N = 152,803) ^*^ Unadjusted weighted obesity prevalence. 95% confidence intervals are indicated by brackets.

**TABLE:** Prevalence of obesity*† among public school children in grades K, 1, 3, 5, and 7, by school year and selected characteristics — Anchorage metropolitan area, 2003–04 through 2010–11 school years (N = 152,803)

Characteristic	2003–04	2004–05	2005–06	2006–07	2007–08	2008–09	2009–10	2010–11	Adjusted p-value for trend§	% change from 2003–04 to 2010–11
							
	% (95% CI)	% (95% CI)	% (95% CI)	% (95% CI)	% (95% CI)	% (95% CI)	% (95% CI)	% (95% CI)		
**No. of students**	16,909	17,506	17,607	18,813	20,202	20,407	20,481	20,878		
**Overall**	16.8 (16.1–17.5)	16.4 (15.8–17.0)	16.2 (15.6–16.8)	16.3 (15.7–16.8)	16.2 (15.7–16.7)	16.9 (16.4–17.4)	16.3 (15.8–16.8)	16.3 (15.8–16.8)	<0.001	−3.0
**Sex**										
Boys	18.1 (17.1–19.0)	18.3 (17.5–19.3)	17.6 (16.7–18.5)	17.6 (16.8–18.4)	17.4 (16.7–18.2)	18.5 (17.7–19.2)	17.8 (17.1–18.5)	17.1 (16.4–17.8)	<0.001	−5.5
Girls	15.4 (14.6–16.4)	14.3 (13.6–15.2)	14.7 (13.9–15.6)	14.8 (14.1–15.6)	14.9 (14.2–15.6)	15.3 (14.6–16.0)	14.7 (14.0–15.4)	15.4 14.7–(16.1)	0.087	0.0
**Race/Ethnicity** ¶										
White, non-Hispanic	13.9 (13.1–14.8)	13.3 (12.6–14.1)	13.0 (12.3–13.8)	13.0 (12.3–13.7)	12.9 (12.2–13.5)	12.3 (11.7–13.0)	11.9 (11.3–12.5)	11.8 (11.2–12.4)	<0.001	−15.1
AI/AN	20.8 (18.9–22.8)	21.2 (19.3–23.2)	21.0 (19.1–22.9)	21.1 (19.4–23.0)	21.8 (20.1–23.6)	21.2 (19.5–23.0)	21.0 (19.3–22.8)	20.5 (18.8–22.3)	0.528	−1.4
All other	22.5 (21.4–23.6)	22.0 (21.0–23.1)	22.0 (21.0–23.0)	22.3 (21.3–23.3)	21.9 (21.0–22.9)	22.7 (21.8–23.7)	22.0 (21.1–22.9)	22.3 (21.4–23.3)	0.870	−0.9
**Grade**										
K, 1, and 3	14.8 (14.0–15.7)	14.2 (13.4–15.0)	14.2 (13.5–15.0)	14.2 (13.5–15.0)	14.4 (13.8–15.1)	14.8 (14.2–15.4)	14.2 (13.6–14.8)	14.0 (13.4–14.6)	0.001	−5.4
5 and 7	19.4 (18.5–20.4)	19.4 (18.5–20.3)	19.1 (18.1–20.1)	19.2 (18.3–20.1)	18.8 (18.0–19.7)	19.9 (19.0–20.8)	19.5 (18.6–20.3)	19.8 (19.0–20.7)	0.075	2.1
**Site**										
Anchorage School District	18.0 (17.4–18.6)	17.3 (16.7–17.9)	17.4 (16.8–18.0)	17.3 (16.7–17.9)	17.3 (16.7–17.8)	18.2 (17.6–18.8)	17.7 (17.1–18.3)	17.6 (17.0–18.2)	<0.001	−2.2
Matanuska-Susitna Borough School District	12.5 (10.6–14.7)	13.2 (11.7–15.0)	12.3 (10.8–14.0)	12.9 (11.7–14.3)	13.3 (12.2–14.4)	12.7 (11.7–13.7)	12.1 (11.2–13.1)	12.4 (11.5–13.3)	0.781	−0.8
**SES** **										
Higher SES	14.6 (13.8–15.4)	14.6 (13.9–15.3)	13.9 (13.2–14.6)	13.6 (13.0–14.3)	13.8 (13.2–14.4)	13.8 (13.2–14.3)	13.4 (12.8–14.0)	13.4 (12.8–13.9)	<0.001	−8.2
Lower SES	21.5 (20.4–22.7)	20.8 (19.7–22.0)	21.2 (20.1–22.3)	22.4 (21.4–23.6)	21.9 (20.9–22.9)	23.4 (22.4–24.4)	22.4 (21.4–23.4)	22.6 (21.6–23.6)	0.843	5.1
**Grade by SES by race/ethnicity**										
**K, 1, and 3**										
**Higher SES**										
White, non-Hispanic	10.1 (8.9–11.5)	9.8 (8.8–11.0)	9.8 (8.8–11.0)	9.0 (8.1–10.0)	9.3 (8.5–10.2)	9.1 (8.3–9.9)	8.6 (7.9–9.4)	8.9 (8.2–9.7)	0.017	−11.9
AI/AN	15.7 (12.1–20.2)	16.7 (13.4–20.6)	16.8 (13.6–20.5)	14.9 (12.1–18.3)	18.3 (15.4–21.7)	16.3 (13.7–19.3)	16.4 (13.8–19.5)	17.2 (14.4–20.3)	0.407	9.6
All other	15.5 (13.7–17.6)	17.4 (15.6–19.4)	16.0 (14.2–18.0)	16.9 (15.3–18.8)	16.5 (14.9–18.2)	14.5 (13.0–16.1)	15.7 (14.2–17.4)	15.5 (14.0–17.1)	0.255	0.0
**Lower SES**										
White, non-Hispanic	18.1 (15.9–20.6)	15.5 (13.5–17.8)	14.8 (12.9–16.8)	16.9 (14.8–19.2)	16.0 (14.0–18.2)	13.5 (11.8–15.5)	13.2 (11.5–15.2)	12.7 (11.0–14.7)	0.001	−29.8
AI/AN	21.3 (18.3–24.7)	19.7 (16.7–23.1)	21.5 (18.5–24.8)	25.3 (22.0–28.8)	25.2 (21.9–28.7)	23.7 (20.4–27.2)	22.3 (19.0–25.9)	21.8 (18.6–25.4)	0.110	2.3
All other	24.0 (22.2–26.0)	22.2 (20.5–24.0)	22.6 (21.0–24.3)	23.5 (21.8–25.2)	23.2 (21.6–24.8)	25.2 (23.5–26.9)	23.8 (22.2–25.5)	22.7 (21.1–24.4)	0.633	−5.4
**Grade by SES by race/ethnicity**										
**5 and 7**										
**Higher SES**										
White, non-Hispanic	16.2 (14.9–17.7)	15.3 (14.1–16.5)	15.0 (13.7–16.4)	15.2 (14.1–16.5)	14.8 (13.7–16.0)	14.1 (13.0–15.3)	14.6 (13.5–15.8)	13.8 (12.8–15.0)	0.003	−14.8
AI/AN	22.6 (18.9–26.8)	25.6 (21.9–29.7)	21.8 (18.2–26.0)	23.6 (20.1–27.5)	21.7 (18.3–25.6)	23.6 (20.1–27.4)	21.3 (18.1–25.0)	18.9 (15.8–22.4)	0.216	−16.4
All other	22.8 (20.7–25.1)	22.3 (20.3–24.5)	21.4 (19.4–23.6)	22.2 (20.2–24.4)	23.5 (21.5–25.6)	22.6 (20.7–24.7)	20.8 (19.0–22.8)	22.9 (20.9–25.0)	0.966	0.4
**Lower SES**										
White, non-Hispanic	17.3 (14.7–20.2)	21.8 (18.6–25.2)	20.9 (17.9–24.2)	22.7 (19.8–25.9)	21.4 (18.8–24.3)	22.3 (19.5–25.4)	20.7 (17.7–24.0)	22.4 (19.4–25.7)	0.067	29.5
AI/AN	25.4 (20.7–30.8)	24.9 (19.5–31.2)	25.7 (20.9–31.1)	20.1 (16.1–24.9)	23.1 (18.8–28.0)	22.6 (18.4–27.4)	26.8 (22.2–32.0)	26.0 (21.6–30.9)	0.982	2.4
All other	29.1 (26.3–32.2)	29.2 (26.1–32.4)	29.8 (27.2–32.7)	28.4 (26.0–31.1)	26.9 (24.4–29.4)	31.4 (28.9–34.1)	29.0 (26.6–31.4)	31.1 (28.7–33.6)	0.332	6.9

**Abbreviations:** CI = confidence interval; AI/AN = American Indian/Alaska Native; SES = socioeconomic status.

* Defined as a body mass index in ≥95th percentile.

† Weighted percentages; unadjusted obesity prevalence.

§ Logistic regression model included a linear term for trend in obesity, adjusted for sex, race/ethnicity, grade, and site.

¶ Race/ethnicity categories are mutually exclusive. All other races/ethnicities category contains Asian/Pacific Islander, black, Hispanic, and multiple races.

** Subsidized lunch is a school-level indicator for the percentage of students in the free and reduced lunch program. High socioeconomic status (SES) schools are those with a low percentage of students in the free and reduced lunch program (0%–50%). Low socioeconomic status schools are those with a high percentage of students in the subsidized lunch program (51%–100%).
